# Prevalence and Management Recommendations for Disease-Related Malnutrition in Chronic Kidney Disease Patients with and without Diabetes

**DOI:** 10.1155/2022/4419486

**Published:** 2022-08-25

**Authors:** Li-Li Dai, Wei-Li Li, Ding-Feng Zheng, Wei-Hong Wang, Hao-Fen Xie, Jian-Wei Ma

**Affiliations:** ^1^Department of Nephrology Department, Ningbo First Hospital, Ningbo 315010, China; ^2^Department of Nursing Department, Ningbo First Hospital, Ningbo 315010, China

## Abstract

**Objective:**

To investigate the nutritional risk, malnutrition, severe malnutrition, and malnutrition prevalence of different stages in chronic kidney disease (CKD) patients with and without diabetes mellitus using the Global Leadership Initiative on Malnutrition (GLIM), and to analyze the causes of malnutrition and to improve the clinical outcomes of patients for early intervention.

**Methods:**

A total of 683 patients with CKD who were hospitalized in our hospital from January 2020 to January 2021 were enrolled and divided into subgroups 1 to 5 according to whether they were complicated with diabetes and glomerular filtration rate. Using the second step of the malnutrition (GLIM) diagnostic tool and 2 previously commonly used malnutrition assessment methods (body mass index <18.5 kg/m^2^ with poor general condition, 3 points for nutritional deficiency in nutritional risk screening), combined with clinical research on the main causes of malnutrition, the intervention measures were discussed.

**Results:**

The prevalence of malnutrition was 16.7% (114/683) in the patients included in the survey using the diagnostic criteria of malnutrition (GLIM) (excluding whole body muscle mass index). The prevalence of malnutrition in CKD patients with and without diabetes was 23.7% and 12.6%, respectively. The overall prevalence rate of severe malnutrition was 14.2%, and the prevalence rates of those with and without diabetes were 19.0% and 11.4%, respectively; the results of the two methods of malnutrition assessment showed that the prevalence of malnutrition in CKD patients with diabetes was higher than that in the uncombined group. There was no severe malnutrition in patients with CKD stages 1 and 2. From CKD stage 3 onwards, the severe malnutrition in the diabetic group was significantly higher than that in the uncombined group.

**Conclusion:**

With the progression of CKD, the incidence of malnutrition also gradually increased, indicating that malnutrition is related to primary diseases and concomitant diseases. Attention should be paid to the malnutrition of CKD patients with diabetes, and clinical medical staff need to pay early attention to various diseases that lead to the progression of CKD, such as diabetes, primary nephropathy, and other factors, to prevent complications and delay the progression of CKD.

## 1. Introduction

A nationwide cross-sectional survey of a representative sample of Chinese adults showed that the prevalence of chronic kidney disease (CKD) in China was about 10.8% [1], and it has been reported in the literature that the incidence of malnutrition in CKD patients ranges from 12% to 40% [2, 3]. Although malnutrition needs to be corrected, this malnutrition cannot be improved by supplementing nutrients because the causes of CKD and the progression of CKD are accompanied by underlying diseases, such as diabetes, hypertension, proteinuria, etc. Malnutrition outcomes can also be indirectly or exacerbated if associated diseases are not well controlled. At present, the first cause of CKD in my country has changed from chronic nephritis to diabetes, that is, some CKD is a complication of diabetes [4]. Disease-related malnutrition (DRM) is considered when the severity or persistence of inflammation results in a loss of lean body mass associated with functional impairment. This form of malnutrition is partly attributable to reduced nutrient intake but is also closely related to the impact of inflammatory states on intermediary metabolism [5]. However, there are not many concepts about disease-related malnutrition at present, and it may be one-sided and misleading that nutritional supplements are an important means of treating malnutrition. Disease-related malnutrition (DRM) is a common clinical problem. It has been reported abroad that chronic kidney disease is the fourth most common diagnosis related to DRM, accounting for 15% [6]. Therefore, this study hopes to summarize the prevalence of malnutrition and severe malnutrition in CKD complicated with diabetes mellitus and different stages of CKD through a cross-sectional survey, and provide suggestions for early CKD intervention measures, delaying CKD progression, and appropriate nutritional supplementation.

## 2. Objects and Methods

### 2.1. Research Objects

1. In this study, the general data of inpatients in the Nephrology Department of Ningbo First Hospital from January 2020 to January 2021 were collected, and a total of 683 cases of CKD patients were selected as the research objects..
2. Inclusion criteria: (1) patients who met the criteria for chronic kidney disease (CKD) defined by the Kidney Diseases Outcome Quality Initiative (K/DOQI) established by the Kidney Foundation International (NKF) in 2002. (2) The length of hospitalization is more than 1 day. (3) The age is 18 to 90 years old, and effective language communication can be carried out [2].
3. Exclusion criteria: (1) those with mental disorders who cannot cooperate or who are critically ill and unable to complete relevant assessments; (2) incomplete data on body weight, diet, and disease conditions in the past 3 months; (3) excluding the possibility of acute renal failure, and having no recent severe heart failure, unstable angina, active liver disease, trauma, surgery, chronic wasting diseases (such as tuberculosis, tumor, and hyperthyroidism), etc. The research protocol was reviewed and approved by the ethics committee of our hospital, and the research was approved.

### 2.2. Research Methods

**Figure 1:**
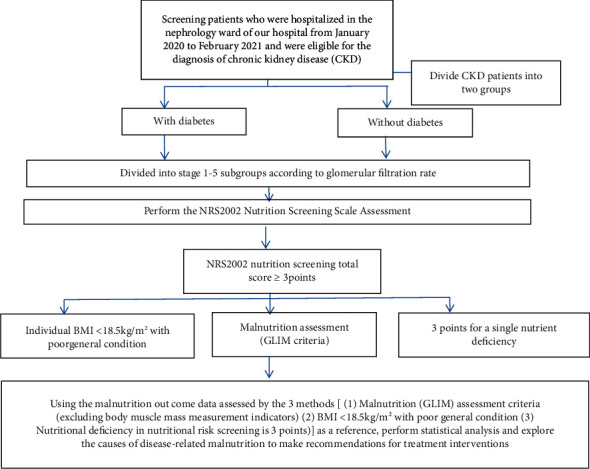
Research flowchart.

Basic information was collected and recorded, including gender, age, height, weight, and past history; the patients were divided into 2 groups with or without diabetes (diagnosed with diabetes), and were divided into subgroups of stages 1 to 5 according to the glomerular filtration rate; nutritional risk screening NRS2002 was carried out, the NRS2002 screening scale was filled, and malnutrition was diagnosed according to the assessment standard of malnutrition (GLIM). At the same time, the enrolled CKD patients were used as a reference by the data of two other malnutrition assessment methods (body mass index < 18.5 kg/m^2^ with poor general condition, nutritional deficiency in nutritional risk screening, 3 points). Research flow chart is shown in Figure 1.

### 2.3. Observation Indicators and Evaluation Criteria

#### 2.3.1. Nutritional Risk Screening

Within 24 hours of admission, patients were screened for nutritional risk by trained nursing staff using the Nutritional Risk Screening NRS 2002. A score of ≥3 indicates nutritional risk and a score of <3 indicates no nutritional risk [7].

#### 2.3.2. Malnutrition Assessment (Diagnosis)

On the basis of a positive nutritional risk screening, malnutrition assessment (diagnosis) was performed within 48 hours of admission.

According to the second step of the malnutrition (GLIM) assessment criteria, at least one of the phenotypic indicators and etiological indicators are met to be diagnosed as malnutrition [8]. Due to the differences in race and gender in the reference standard of muscle mass, and the lack of accurate and popular measurement conditions in China, there is no reliable large-sample normal value and threshold reference, so this study used BMI and involuntary weight loss. Phenotypic indicators are not included in the measurement of muscle mass. CKD staging was based on the recommendations of the American KDOQI expert staging method.

At the same time, two sources of malnutrition assessment data were used as a reference: (1) BMI < 18.5 kg/m2, with poor general clinical observation [9], and (2) the nutritional status part of the NRS2002 score ≥3 points.

### 2.4. Quality Control

Referring to CSPEN's “Clinical Diagnosis and Treatment Guidelines - Parenteral and Enteral Nutrition Volume (2008 Edition),” the “nutritional screening-undernutrition” established by the Chinese Medical Association Parenteral and Enteral Nutrition Society-support outcome cost/effectiveness ratio, NUSOC) Multicenter Data Sharing Collaborative Group” research results as preinvestigation training content to conduct unified training for investigators [10].

Definition of statistical power: the probability of correctly rejecting H0 (probability of not making type II error).

### 2.5. Statistical Methods

SPSS 19.0 statistical software was used for analysis, normally distributed measurement data were expressed as mean ± standard deviation, using the *t*-test; count data were compared using *χ*^2^ test; *P* < 0.05 was considered statistically significant.

## 3. Results and Discussion

### 3.1. General Information of CKD Patients with or without Diabetes

A total of 683 CKD hospitalized patients were included, including 433 males and 250 females, aged 60.7 ± 16.6 years, 253 CKD patients with diabetes (37.04%), and 430 CKD patients without diabetes (62.96%). Stage 3 and above patients accounted for 85.2% (see Table 1 for details).

### 3.2. Nutritional Risk in CKD Patients with Different Stages of Diabetes Mellitus

Among the 683 CKD patients, the overall prevalence of nutritional risk was 25.2% (172/683), and the prevalence of nutritional risk in CKD patients with and without diabetes was 33.2% (84/253) and 20.5% (88/430), respectively. The difference between the two groups was statistically significant (*χ*^2^ = 13.71, *P* < 0.05). The nutritional risk prevalence rates of CKD stage 1–5 patients with diabetes were 5.6%, 16.7%, 16.1%, 32.7%, and 50% (*χ*^2^ = 29.754, *P* < 0.05), and nutritional risk prevalence rates of CKD stage 1–5 patients without diabetes were 5.1%, 6.3%, 11.6%, 13.8%, and 36.4%, respectively (*χ*^2^ = 41.316, *P* < 0.05). Among them, the prevalence of nutritional risk increased significantly in CKD stages 2 and 3, respectively, with or without diabetes (see Table 2 for details).

### 3.3. Malnutrition in CKD Patients with Different Stages of Diabetes Mellitus

The overall prevalence of malnutrition was 16.7% (114/683), and the prevalence of malnutrition in CKD patients with and without diabetes was 23.7% and 12.6% (60/253, 54/430), respectively. The difference between the two groups was statistically significant (*χ*^2^ = 14.260, *P* < 0.05). The prevalence of malnutrition in CKD stages 1 to 5 with diabetes was 5.6%, 8.3%, 16.1%, 21.8%, and 33.9%, respectively; the prevalence of CKD stage 1 to 5 without diabetes was 2.8%, 3.1%, 5.4%, 10.7%, and 23%. At the same time, the poor general condition of clinical observation and the 3-point assessment of nutritional deficiency in the NRs 2002 scale were recorded as BMI < 18.5 kg/m^2^, and the prevalence of malnutrition was 13.5% (92/683), 18.7% (128/683)). The results of malnutrition assessment and diagnosis from the three sources showed that the prevalence of malnutrition in patients with CKD with diabetes was significantly higher than that in patients with CKD without diabetes, and with the decrease of eGFR, the prevalence of malnutrition also gradually increased. CKD stage 3 malnutrition was significantly higher regardless of diabetes, as shown in Table 3.

### 3.4. Severe Malnutrition in CKD Patients with Different Stages of Diabetes Mellitus

The overall prevalence of severe malnutrition in CKD patients was 14.2% (97/683), and the prevalence of severe malnutrition in CKD patients with and without diabetes was 19% and 11.4% (48/253, 49/430), respectively. There was a statistically significant difference between the two groups (*χ*^2^ = 7.505, *P* < 0.05). The prevalence of severe malnutrition in CKD stages 1 to 5 with diabetes was 0%, 0%, 9.7%, 18.2%, and 30.2%, respectively; the prevalence of severe malnutrition in CKD stages 1 to 5 in diabetes was 0%, 0%, 5.4%, 9.2%, and 21.8%, respectively. Severe malnutrition occurred since CKD stage 3 in the group with or without diabetes (see Table 4 for details).

## 4. Discussion

### 4.1. Nutritional Risk in CKD Patients with Diabetes Appears Earlier and Is Related to CKD Stage

As shown in Table 2, the prevalence of nutritional risk in CKD patients was 25.2% (172/683), and the prevalence of nutritional risk with and without diabetes was 33.2% (84/253) and 20.5% (88/430), respectively; the difference between the groups was statistically significant (*χ*^2^ = 13.71, *P* < 0.05). The nutritional risk of the same CKD stage was similar except for stage 1, and the prevalence of the other stages was higher in the diabetic group, which suggested that CKD patients with diabetes mellitus were more prone to nutritional risks. The nutritional risk is 11.0% in CKD stages 1–3 and 35.8% in stages 4–5. The results of the study are similar to those of Li Jing et al. [2]. Nutritional risk is related to the CKD stage; that is, as eGFR decreases in CKD patients, the prevalence of nutritional risk increases. The prevalence of nutritional risk in the nondiabetic group increased significantly from the 3rd stage of CKD, while the diabetes group increased significantly in the 2nd stage, indicating that the nutritional risk of the diabetic patients appeared earlier in the course of CKD. Up to 37% of CKD patients have diabetes. Therefore, CKD patients with diabetes should pay more attention to their nutritional status early, to achieve early detection, early prevention, and early intervention, so as to improve their nutritional status and help to delay the progress of CKD.

### 4.2. CKD Patients with Diabetes Are Malnourished and Have a High Prevalence of Severe Malnutrition

In this study, the overall prevalence of malnutrition was 16.7% (114/683) diagnosed by the assessment criteria of malnutrition (GLIM). The prevalence of malnutrition with or without diabetes was 23.7% (60 / 253) and 12.6% (54 / 430). From Table 4, it was found that the total prevalence of severe malnutrition was 14.2%, and the prevalence of severe malnutrition in the group with or without diabetes was 19.0% (48/253) and 11.4% (49/430), suggesting that CKD patients with diabetes are more prone to severe malnutrition. There was no significant difference in the prevalence of malnutrition between the two groups in CKD stages 1 and 2, and no severe malnutrition occurred; as the eGFR of patients decreased, the prevalence of severe malnutrition in CKD stage 3 increased exponentially, and the same stage with diabetes mellitus group of severe malnutrition was significantly higher than that in the uncombined group. In addition, the prevalence rates of severe malnutrition in CKD stages 3 to 5 were 6.8%, 13.3%, and 25.1%, respectively, which is in line with the report [11] that found that the severe malnutrition in CKD stages 3 and 4 was assessed by subjective global assessment (SGA). The results were similar, and its prevalence also increased as eGFR decreased in CKD patients.

### 4.3. CKD Malnutrition Belongs to Disease-Related Malnutrition (DRM) and Should Be Intervened as Soon as Possible

Zhang et al. [12] statistics show that since 2011, diabetes has surpassed glomerulonephritis to become the first cause of chronic kidney disease (CKD) among hospitalized patients in urban residents in my country. Metabolic disorders caused by hyperglycemia cause inflammatory disease, and the inflammatory factor interleukin-8 can increase glomerular permeability and produce proteinuria [13]. The results of this study showed that CKD patients with diabetes were more prone to malnutrition, and the prevalence of severe malnutrition was significantly higher than that of the uncombined group; with the progression of the disease, the prevalence of malnutrition in CKD stage 3 increased exponentially, and the same stage The severe malnutrition in the diabetic group was significantly higher than that in the uncombined group; from this point of view, the malnutrition in CKD fits the concept of DRM. Sun Shilan reported [14] that the pathophysiological factors affecting the progression of CKD include (1) activation of the renin-angiotensin system (RAS) in the kidney; (2) hypertension; (3) proteinuria; (4) anemia; (5) metabolic acidosis; and (6) calcium and phosphorus metabolism disorders. Therefore, treatment of the above factors can delay the progression of CKD.

Studies have also shown that [15, 16] a variety of therapies and drugs can reduce urinary protein excretion and improve glomerular sclerosis in patients with diabetic nephropathy, thereby protecting kidney function and delaying the progression of kidney disease. Dysbiosis of intestinal flora plays an important role in malnutrition in diabetic CKD. Malnutrition in diabetic CKD patients is closely related to the imbalance of intestinal microbial communities, and metabolic wastes in the blood cannot be fully excreted through the kidneys and enter the intestinal lumen, causing disturbance of the microbiota in the intestinal tract and resulting in a decrease in the number of probiotics [17, 18]. Toxic metabolites produced by gut microbes rapidly increase, increasing the rate of renal deterioration. Stabilization of the intestinal flora in the body will help protect the kidneys, but if the intestinal flora is out of balance, it will cause kidney disease. The results of this study found that CKD patients with diabetes had a higher prevalence of malnutrition than the group without diabetes. Therefore, it is very important to strengthen nutrition to prevent complications and delay CKD, especially in diabetic patients. A number of studies have shown that the effective use of high-quality low-protein diet can play a significant role in the control of blood glucose levels in patients with diabetic nephropathy, so as to effectively control the development of the disease, significantly improve the malnutrition status and laboratory indicators, and ultimately achieve an effective prognosis for patients with diabetic nephropathy [19–21].

There are still some limitations in this study. Since the subjects of this study are mainly CKD patients with diabetes and without diabetes, the malnutrition of other CKD patients with different comorbidities has not been studied in depth, nor is it a multicenter, large-sample study, and it is necessary to collect larger samples in the future for related research prospective study. In addition, this study has not yet analyzed the influencing factors of malnutrition in CKD patients, the mechanism of malnutrition and intestinal microbial imbalance in CKD patients, and more in-depth analysis will be conducted in future studies.

## 5. Conclusions

In conclusion, DRM in CKD patients is positively correlated with CKD stage and diabetes. The Chinese Clinical Practice Guidelines for Nutritional Treatment of Chronic Kidney Disease [22] proposed that reasonable energy intake is the premise to ensure the nutritional supply of CKD patients, and recommended the principles of a protein-containing diet and combined supplementation of ketoacid preparations for CKD patients of different stages. Nutrients have a certain role in improving the nutritional status of patients, but they are also one-sided; the 2020 KDIGO guideline [23] pointed out that patients with CKD and diabetes are often accompanied by multisystem diseases and require multidisciplinary professional medical team treatment. These patients are high-risk groups for CKD progression. It should be included in the comprehensive management of the whole course of the disease early. Comprehensive management includes lifestyle intervention, risk management (lowering blood sugar, blood pressure, blood lipid control, etc.), and drug treatment; developing good living habits, a balanced diet, and scientific exercise are all effective measures.

## Figures and Tables

**Table 1:** General data of CKD patients with or without diabetes (*x* ± *s* (*n*)).

CKD classification	Sex		Age	BMI	Clinical stage				
	Male	Female	(years)	(kg/m^2^)	I	II	III	IV	V
With diabetes (*n* = 253)	166	87	65.8 ± 12.7	23.6 ± 4.3	18	12	62	55	106
Without diabetes (*n* = 430)	267	163	57.8 ± 17.9	23.1 ± 3.9	39	32	129	65	165
Total (683)	433	250	60.7 ± 16.6	23.2 ± 4.1	57	44	191	120	271

**Table 2:** Prevalence of nutritional risk (NRS ≥ 3 points) in CKD patients with or without diabetes in different stages (*n* (%)).

CKD classification	With diabetes *n* = 253	Without diabetes *n* = 430	*χ * ^2^	*P value*
Stage I	5.6 (1/18)	5.1 (2/39)	0.005	1.0
Stage II	16.7 (2/12)	6.3 (2/32)	1.146	0.297
Stage III	16.1 (10/62)	11.6 (15/129)	0.746	0.495
Stage IV	32.7 (18/55)	13.8 (9/65)	6.091	0.014^*∗*^
Stage V	50 (53/106)	36.4 (60/165)	4.937	0.026^*∗*^
Total	33.2 (84/253)	20.5 (88/430)	13.71	0.00^*∗*^
*χ * ^2^	29.754	41.316		
*P value*	0.00^*∗*^	0.00^*∗*^		

^*∗*^ *P* < 0.05.

**Table 3:** Malnutrition in CKD patients with or without diabetes in different stages (*n* (%)).

CKD classification	Stage	*n*	Different methods for assessing malnutrition			*χ * ^2^	*P value*
			GLIM step 2 criteria for diagnosing malnutrition	Low BMI and poor general assessment of malnutrition	NRS2002 nutrition deficiency part reaches 3 points		
With diabetes	I	18	1 (5.6%)	1 (5.6%)	1 (5.6%)	0.432	1
	II	12	1 (8.3%)	1 (8.3%)	2 (16.6%)	0.731	1
	III	62	10 (16.1%)	6 (9.7%)	7 (11.2%)	1.29	0.616
	IV	55	12 (21.8%)	7 (12.7%)	8 (14.5%)	3.83	0.171
	Stage V and dialysis	106	36 (33.9%)	30 (28.3%)	37 (35%)	1.24	0.567
	Total	253	60 (23.7%)	45 (17.8%)	55 (21.7%)	2.796	0.249

Without diabetes	I	39	1 (2.8%)	0 (0%)	1 (2.8%)	1.259	1
	II	32	1 (3.1%)	1 (3.1%)	2 (6.2%)	0.685	1
	III	129	7 (5.4%)	7 (5.4%)	10 (7.8%)	0.8	0.775
	IV	65	7 (10.7%)	6 (9.2%)	9 (13.8%)	0.717	0.781
	Stage V and dialysis	165	38 (23%)	33 (20%)	51 (30.9%)	5.635	0.062
	Total	430	54 (12.6%)	47 (10.9%)	73 (17.0%)	7.03	0.03^*∗*^

Total		683	114 (16.7%)	92 (13.5%)	128 (18.7%)	7.138	0.028^*∗*^
*χ * ^2^			14.260	4.424	2.372		
*P value*			0.00^*∗*^	0.015^*∗*^	0.129		

^*∗*^ *P*<0.05 (statistically different).

**Table 4:** Prevalence of severe malnutrition in CKD patients with or without diabetes in different stages (*n* (%)).

CKD classification	With diabetes *n* = 253	Without diabetes *n* = 430	*χ * ^2^	*P value*
I	0 (0/18)	0 (0/39)	—	—
II	0 (0/12)	0 (0/32)	—	—
III	9.7 (6/62)	5.4 (7/129)	1.193	0.275
IV	18.2 (10/55)	9.2 (6/65)	2.066	0.151
V	30.2 (32/106)	21.8 (36/165)	2.406	0.121
Total	19.0 (48/253)	11.4 (49/430)	7.505	0.006^*∗*^
*χ * ^2^	19.206	31.738		
*P value*	0.001^*∗*^	0.00^*∗*^		

^*∗*^ *P* < 0.05 (statistically different).

## Data Availability

All data generated or analyzed during this study are included in this published article.
